# Comparison of diabetes distress and depression screening results of emerging adults with type 1 diabetes onset at different ages: findings from the German early-onset T1D study and the German Diabetes Study (GDS)

**DOI:** 10.1186/s13098-023-00994-2

**Published:** 2023-02-19

**Authors:** Anna Stahl-Pehe, Christina Bächle, Kálmán Bódis, Oana-Patricia Zaharia, Karin Lange, Reinhard W. Holl, Michael Roden, Joachim Rosenbauer, for the GDS Group, M. Roden, H. Al-Hasani, B Belgardt, GJ. Bönhof, V Burkart, A. E. Buyken, G. Geerling, C. Herder, A. Icks, K. Jandeleit-Dahm, J. Kotzka, O. Kuß, E. Lammert, W. Rathmann, V. B. Schrauwen-Hinderling, J. Szendroedi, S. Trenkamp, R. Wagner

**Affiliations:** 1 Institute for Biometrics and Epidemiology, German Diabetes Center (DDZ), Leibniz Center for Diabetes Research at Heinrich Heine University Düsseldorf, Auf’m Hennekamp 65, 40225 Düsseldorf, Germany; 2 German Center for Diabetes Research (DZD), Munich-Neuherberg, Germany; 3 Institute for Clinical Diabetology, German Diabetes Center (DDZ), Leibniz Center for Diabetes Research at Heinrich Heine University Düsseldorf, Düsseldorf, Germany; 4 Department of Endocrinology and Diabetology, Medical Faculty and University Hospital, Heinrich Heine University, Düsseldorf, Germany; 5 Medical Psychology Unit, Hannover Medical School, Hannover, Germany; 6 Institute of Epidemiology and Medical Biometry, ZIBMT, University of Ulm, Ulm, Germany

**Keywords:** Type 1 diabetes, Epidemiology, Diabetes distress, Depression, Screening

## Abstract

**Background:**

Diabetes distress is increasingly considered one of the most important psychosocial issues in the care of people with type 1 diabetes (T1D). We analyse whether diabetes distress and depression screening results of emerging adults are associated with the age at T1D onset.

**Methods:**

Data were taken from two cohort studies conducted at the German Diabetes Center, Düsseldorf, Germany. The 18–30-year-old participants had an age at onset either before the age of 5 years (childhood-onset long-term T1D study group, N = 749) or during adulthood (adult-onset short-term T1D study group from the German Diabetes Study (GDS), N = 163). Diabetes distress and depression screening were analysed by means of the 20-item Problem Areas in Diabetes (PAID-20) scale and the nine-item depression module from the Patient Health Questionnaire (PHQ-9). The average causal effect of age at onset was estimated by a doubly robust causal inference method.

**Results:**

The PAID-20 total scores were increased in the adult-onset study group [potential outcome mean (POM) 32.1 (95% confidence interval 28.0; 36.1) points] compared to the childhood-onset study group [POM 21.0 (19.6; 22.4) points, difference 11.1 (6.9; 15.3) points, p<0.001] adjusted for age, sex and haemoglobin A1c (HbA1c) levels. Moreover, more participants in the adult-onset group [POM 34.5 (24.9; 44.2) %] than in the childhood-onset group [POM 16.3 (13.3; 19.2) %] screened positive for diabetes distress [adjusted difference 18.3 (8.3; 28.2) %, p<0.001]. The PHQ-9 total score [difference 0.3 (-1.1; 1.7) points, p=0.660] and the proportion of participants with a positive screening result for depression [difference 0.0 (-12.7; 12.8) %, p=0.994] did not differ between the groups in the adjusted analyses.

**Conclusions:**

Emerging adults with short-term type 1 diabetes screened positive for diabetes distress more often than adults with type 1 diabetes onset during early childhood when age, sex and HbA1c values were considered confounding factors. Accounting for age at onset or the duration of diabetes may help explain the heterogeneity in the data when psychological factors are examined.

**Supplementary Information:**

The online version contains supplementary material available at 10.1186/s13098-023-00994-2.

## Background

Type 1 diabetes (T1D) poses a particular challenge during emerging adulthood, the life stage from age 18 to 30, when many emotional, social, and developmental changes occur [1]. Data from qualitative studies have shown the complexity of the emotional burden of living with T1D as an emerging adult [2, 3]. The emotional burden of the multiple daily diabetes management tasks within a social and developmental context, frustrations, and worries that result from living with T1D are captured in the concept of “diabetes distress”. Thus, diabetes distress is not a psychopathology, but it is viewed as part of the spectrum of diabetes [4]. As an adaptive emotional response to disease burden, diabetes distress is a diabetes-specific concept with some overlap with depression [5, 6]. Diabetes distress is increasingly considered one of the most important psychosocial issues in the care of people with T1D; therefore, an international consensus study recommended that the measurement of diabetes-related burden or stress should be a key clinical outcome measure in studies of emerging adults [7]. Diabetes distress in emerging adults is still an understudied phenomenon, even though it is common and occurs more frequently in this age- group and is suspected to contribute to adverse outcomes [2], such as higher haemoglobin A1c (HbA1c) levels [8–10], higher glycaemic variability [11], impaired quality of life [9], more severe depressive symptoms [8], and worse physical and mental health [8, 9]. Since diabetes distress, depressive symptoms and HbA1c levels are interrelated, it seems appropriate to examine these factors simultaneously [6, 8, 12].

A systematic review [2] reported that a sample of emerging adults with diabetes onset [8] at a very young age had a much lower prevalence of elevated diabetes distress scores than other samples, possibly related to likely different experiences with T1D. Age at disease onset is a rarely studied risk factor for T1D complications. Findings suggest that early disease onset is a nonmodifiable risk factor for survival [13], cardiovascular outcomes [13], nephropathy [14] and cognitive impairment [15] and that distinct endotypes of T1D related to age at diagnosis exist [16]. To date, the possible relationship between age at onset and psychosocial issues is largely unexplored. In a 14–25-year-old sample, diabetes distress was not correlated with age at onset of diabetes [17]. In another study among older adults, women with childhood-onset T1D had higher regimen-related distress than women with adult-onset T1D [18], while women with adult-onset T1D reported higher dejection [19]. We are not aware of any study that has compared diabetes distress and depression screening results in samples of 18–30-year-old adults with disease onset in childhood and emerging adulthood.

The study aimed to compare two samples of 18- to 30-year-old participants with distinctly different ages at T1D onset and thus different diabetes durations. Our hypothesis was that the two study groups with either early childhood-onset (long-term) T1D or adult-onset (short-term) T1D would differ in their diabetes distress and depression screening results.

## Methods

### Data sources

Data were taken from two cohort studies conducted at the German Diabetes Center (DDZ) in Düsseldorf, Germany. One of these studies was the “Clinical Course of Type 1 Diabetes in Children, Adolescents and Young Adults with Disease Onset at Preschool Age” observational study (childhood-onset study group). The study was conducted in accordance with the principles of the Declaration of Helsinki and was approved by the ethics committee of the University of Düsseldorf (reference number: 3254). All study participants gave written informed consent. The study design of the first questionnaire survey has been described previously [20], and subsequent surveys were designed similarly. In short, nationwide surveys were conducted in the form of standardised self-administered questionnaires. All study participants had a clinical diagnosis of T1D during their first five years of life and a diabetes duration of at least ten years. Details on the cohort study are given in Additional file 1: Figure S1. For this analysis, data from follow-up surveys conducted in 2012–2013, 2015–2016, and 2018–2019 were used because data on diabetes distress were collected only during these years.

The other study was the German Diabetes Study (GDS), which was a prospective observational cohort study that was initiated at the DDZ and developed into a national multicentre study (Additional file 1: Table S1). This study was conducted in accordance with the Declaration of Helsinki and approved by the Ethics Committee of the University of Düsseldorf (reference number: 4508) and has been registered with Clinicaltrials.gov (identification number: NCT01055093). The GDS study design was characterised by intensive phenotyping at 5-year intervals and annual telephone interviews. The primary inclusion criterion was a clinical diagnosis of type 1 or type 2 diabetes mellitus within the last 12 months in adults aged ≥ 18 years (adult-onset study group) according to the current recommendations by the American Diabetes Association. The diagnosis of type 1 diabetes was based on diabetes manifestation with ketoacidosis or immediate insulin requirement along with the presence of at least one islet cell-directed autoantibody or C peptide levels below the detection limit. The exclusion criteria at baseline were poor glycaemic control (HbA1c level > 9.0%), severe diseases and psychiatric disorders, among other criteria, which are described elsewhere in detail [21]. For this analysis, data from the full tests conducted from November 2010 to the end of 2021 were eligible because data on diabetes distress were not collected previously.

### Study population

Emerging adulthood was defined, as usual, as 18 to 30 years of age [22–25]. Thus, study participants aged 18–30 years were eligible for inclusion in this investigation. A total of 821 participants in the childhood-onset study and 195 participants in the GDS fulfilled this criterion. After the exclusion of persons who were not followed up after diabetes distress was included in the questionnaires, 749 participants were included in the childhood-onset study group (T1D onset in 1993–2005, surveyed in 2012–2019) (Additional file 1: Figure S1), and 163 participants were included in the adult-onset study group (T1D onset in 2007–2020, surveyed in 2010–2021) (Additional file 1: Figure S2 and Table S1). The data from each participant’s most recent follow-up were used.

### Variables

The primary outcome was diabetes distress, which was assessed using the 20-item Problem Areas in Diabetes (PAID-20) scale [26]. The total score ranged from 0–100, with higher scores indicating more severe diabetes distress. Following common practice, a total score of 40 or more was considered to indicate seriously increased diabetes distress (screening positive) [27]. The nine-item depression module from the full Patient Health Questionnaire (PHQ-9) was used as a diagnostic algorithm for the screening of depression. The PHQ-9 total score (ranging from 0–27) was used as a continuous measure, with higher scores indicating more severe depressive symptoms. According to the standard cut-off, a total score of 10 or more was considered indicative of depression (screening positive) [28, 29]. If only one item was not answered, the missing value was replaced by the mean value of the completed items according to Kroenke et al. [30] (N = 18 with childhood onset and N = 1 with adult onset). The participants of both study groups answered the printed questionnaires at home. Further demographic and health- and diabetes-related data were considered (Table 1). Height and weight for the calculation of body mass index (BMI) and HbA1c levels were self-reported by the childhood-onset study group but sampled and analysed in a standardised way at the study centre for the adult-onset study group.

**Table 1:** Characteristics of the study groups

Characteristic	Childhood-onset study group		Adult-onset study group		P*
	n	Percent or mean (SD)	n	Percent or mean (SD)	
Sex					0.004
Male	307	41.0	87	53.4	
Female	442	59.0	76	46.6	
Age [years]	749	22.4 (3.1)	163	26.5 (3.2)	< .001
Nationality					0.010
German	728	98.0	158	96.9	
Other	5	0.7	5	3.1	
German and other	10	1.4	0	0	
Employment					
Not employed, training, federal volunteer service, time off (maternity/parental leave)	492	66.1	58	35.6	< .001
Employed part-time/hourly	65	8.7	20	12.3	
Employed full time	187	25.1	85	52.2	
School-leaving certificate					0.036
High school graduation	378	50.5	97	59.5	
Other^a^	371	49.5	66	40.5	
Diabetes passport use					0.197
Yes	281	37.5	70	42.9	
No^b^	468	62.5	93	47.1	
Age at onset [years]	749	3.2 (1.1)	163	24.2 (3.6)	< .001
Diabetes duration [years]	749	19.1 (3.2)	163	2.3 (3.1)	< .001
BMI	739	24.4 (3.9)	162	24.6 (4.6)	0.876
HbA1_c_ [mmol/mol]	701	62 (15)	161	48 (10)	< .001
HbA1_c_ [%]	701	7.8 (1.4)	161	6.6 (0.9)	
PAID-20 total score	724	21.8 (17.9)	145	23.9 (17.1)	0.099
Diabetes distress screening					0.733
Negative (PAID-20 score < 40)	613	82.6	123	81.5	
Positive (PAID-20 score ≥ 40)	129	17.4	28	18.5	
PHQ-9 total score	745	5.7(4.8)	152	4.6 (3.9)	0.025
Depression screening					0.085
Negative (PHQ-9 score < 10)	605	81.1	133	87.5	
Positive (PHQ-9 score ≥ 10)	141	18.9	19	12.5	

^a^including missing data (N = 1 with childhood onset and N = 37 with adult onset)

^b^including missing data (N = 11 with childhood onset and N = 48 with adult onset)

^*^P value of the Wilcoxon test or the chi-squared test, as appropriate, for the comparison of the childhood-onset and adult-onset study groups

### Data analysis

All variables are described as percentages or means and standard deviations (SDs). For the unadjusted comparisons of the two study groups, the Wilcoxon test was applied for continuous variables, and the chi-squared test was used for categorical variables.

We first compared outcome levels between the two study groups in an unadjusted regression model (Model M0, linear model for continuous outcomes (PAID-20 total score, PHQ-9 total score)), and binary outcomes were compared using a logistic model (screening for diabetes distress and depression). To consider confounding, we applied a doubly robust causal inference approach, which is an inverse probability weighted regression adjustment method that combines inverse probability weighting and regression adjustment to estimate potential outcome means [31]. Within this approach, first, a multivariable logistic model (“treatment/exposure model”) is used to estimate the probability of belonging to the exposure (adult-onset T1D) or control group (childhood-onset T1D), dependent on potential confounders of the exposure-outcome relationship. Standardised differences were used to check the balance between groups regarding confounders after inverse probability weighting of the data. Second, a multivariable model (a linear model for continuous outcomes and a logistic model for binary outcomes) with the outcome as the dependent variable (“outcome model”) is applied, including the exposure and potential confounders, and the inverse probability weights estimated from the exposure model are used to estimate regression coefficients. This approach is said to be doubly robust because it provides unbiased estimates for potential outcome means as long as one of the models is correctly specified [32]. Sex and age were considered potential confounders in Model M1; HbA1c level and education level were additionally considered in Model 2 and Model 3, respectively. The results are presented as adjusted potential outcome means (POM) and respective differences (average causal effect, ACE) with 95% confidence intervals (CIs) and respective p values from a Wald test.

All analyses were performed in complete-case analysis, as the rates of missing values of all variables were at most 5%. Two-sided p values < 0.05 were considered statistically significant. All analyses were performed using SAS version 9.4 (SAS Institute Inc., Cary, North Carolina, USA, 2016).

## Results

The study population consisted of 912 adults younger than 31 years of age, with 749 and 163 having childhood-onset (long-term) or adult-onset (short-term) T1D, respectively. The childhood-onset sample had a mean age at onset of 3.2 years and a T1D duration of 13.3–26.6 years. The adult-onset study group had a mean age at onset of 24.2 years and a T1D duration of 0.1–11.1 years. Compared to the childhood-onset study group, the adult-onset study group was characterised by a higher proportion of men, an older mean age, a lower mean HbA1c value, a higher proportion of participants who were employed full-time, and a higher proportion of participants who graduated from high school (Table 1).

### Diabetes distress screening

The crude PAID-20 total score and the proportion of participants who screened positive for diabetes distress were similar in both study groups (Table 1). The crude PAID-20 total score difference with the 95% CI including zero indicated that the ratings did not differ between the childhood-onset and adult-onset cohorts. However, considering differences in the age and sex distribution between the childhood-onset and adult-onset cohorts, the adjusted CI estimate of the ACE suggested that the PAID-20 total scores were 5 (95% CI 1, 10) points higher in the adult-onset group than in the childhood-onset group. Additionally, when differences in HbA1c values were considered, the mean difference in PAID-20 total scores increased to 11 (95% CI 7, 15) points (Table 2). The additional inclusion of education in the model did not change this result.

**Table 2:** Doubly robust estimates of potential outcome means and average causal effects

Outcome	Study group	Model 0			Model 1			Model 2			Model 3		
		n	Mean (CI) or Difference (CI)^a^	P	n	POM (CI) or ACE (CI)^b^	P	n	POM (CI) or ACE (CI)^b^	P	n	POM (CI) or ACE (CI)^b^	P
PAID-20 total score	Adult-onset	145	23.9 (21.0, 26.7)		145	26.5 (22.1, 30.8)		144	32.1 (28.0, 36.1)		144	32.0 (27.9, 36.1)	
	Childhood-onset	724	21.8 (20.5, 23.1)		724	21.4 (20.1, 22.7)		682	21.0 (19.6, 22.4)		682	21.0 (19.6, 22.3)	
	Difference	869	2.1 (-1.1, 5.2)	0.195	869	5.1 (0.5, 9.6)	0.028	826	11.1 (6.9, 15.3)	< 0.001	826	11.0 (6.8, 15.3)	< 0.001
Positive screening for diabetes distress (%)	Adult-onset	151	18.5 (12.3, 24.7)		151	21.4 (11.6, 31.1)		150	34.5 (24.9, 44.2)		150	33.5 (24.4, 42.6)	
	Childhood-onset	742	17.4 (14.7, 20.1)		742	16.9 (14.2, 19.6)		697	16.3 (13.3, 19.2)		697	16.3 (13.4, 19.2)	
	Difference	893	1.2 (-5.6, 7.9)	0.738	893	4.5 (-5.7, 14.6)	0.387	847	18.3 (8.3, 28.2)	< 0.001	847	17.2 (7.8, 26.7)	< 0.001
PHQ-9 total score	Adult-onset	152	4.6 (4.0, 5.2)		152	5.2 (4.4, 6.1)		151	5.9 (4.5, 7.2)		151	5.8 (4.6, 7.0)	
	Childhood-onset	745	5.7 (5.3, 6.0)		745	5.6 (5.3, 6.0)		698	5.5 (5.2, 5.9)		698	5.6 (5.2, 5.9)	
	Difference	897	-1.1 (-1.8, -0.4)	0.003	897	-0.4 (-1.3, 0.5)	0.383	849	0.3 (-1.1, 1.7)	0.660	849	0.3 (-1.0, 1.5)	0.653
Positive screening for depression (%)	Adult-onset	151	12.6 (7.3, 17.9)		151	14.6 (5.8, 23.4)		151	17.2 (4.7, 29.6)		151	16.5 (4.9, 28.0)	
	Childhood-onset	727	18.4 (15.6, 21.3)		727	17.9 (15.0, 20.7)		699	17.1 (14.1, 20.1)		699	17.5 (14.5, 20.5)	
	Difference	878	-5.8 (-11.8, 0.1)	0.056	878	-3.3 (-12.5, 6.0)	0.491	850	0.0 (-12.7, 12.8)	0.994	850	-1.0 (-13.0, 10.9)	0.878

Model 0: not adjusted. Model 1: adjusted for age and sex. Model 2: adjusted for age, sex and HbA1c level. Model 3: adjusted for age, sex, HbA1c level and school-leaving certificate

^a^Unadjusted mean and 95% confidence interval (CI) with robust variance estimation

^b^Estimate and 95% confidence interval of the potential outcome mean (POM) in the adult-onset and childhood-onset study groups and the respective average causal effect (ACE, difference of the adult-onset and childhood-onset POMs)

The proportion of participants who screened positive for diabetes distress was similar in both study groups according to the crude difference and the ACE after adjusting for age and sex. However, in Model 2, our results suggested that more emerging adults (18%, 95% CI 8%, 28%) in the adult-onset study group than in the childhood-onset study group screened positive for diabetes distress, and Model 3 confirmed the result (Table 2).

Considering the individual PAID-20 items, the groups differed significantly in four of the 20 items. Study participants in the adult-onset group felt particularly burdened regarding eating (Items 5 and 11), and they felt more scared when thinking about living with diabetes (Item 3). Feelings of guilt or anxiety when getting off track with diabetes management (Item 13) was a more important issue for participants in the childhood-onset study group (Additional file 1: Table S2).

### Depression screening

The PHQ-9 total score was slightly higher in the childhood-onset group than in the adult-onset group (Table 1). The crude difference of 1 point in the PHQ-9 total score disappeared after adjustment for additional variables (Table 2). According to the chi-squared test and the doubly robust estimations, the proportion of participants who screened positive for depression did not differ between the groups.

## Discussion

The present study is the first to compare diabetes distress and depression screening results in emerging adults with significantly different ages at T1D onset. The main finding of our study is that emerging adults with a short T1D duration had higher PAID-20 total scores and screened positive for diabetes distress more often than adults with long-term T1D, considering relevant confounders. However, the two study groups did not differ regarding the depression screening results.

### Comparison with previous literature

Roughly estimated, 20% to 40% of all people with diabetes are considered to experience distress, with differentially high prevalence rates attributable to sample characteristics, such as diabetes type, disease duration, age, sex, and ethnicity [33]. The two groups of participants examined in this study had a crude proportion of 17–18% of participants who screened positive for diabetes distress. However, considering influencing factors, in particular, HbA1c levels, it became apparent that the adjusted diabetes distress prevalence in emerging adults with adult-onset (short-term) T1D was approximately 18% higher than that in their peers with childhood-onset (long-term) T1D. The marked impact of HbA1c was in line with previous studies that observed diabetes distress to be associated with higher HbA1c values [33]. In addition, our results are consistent with previous studies that have shown that diabetes distress is more strongly associated with HbA1c than depressive symptoms in adolescents [34] and adults [35, 36] with T1D. The lower HbA1c value in the adult-onset study group can be explained not only by the selection criteria (HbA1c ≤ 9%) but also by the fact that some of the volunteers may have been in remission. Data from most subjects in the adult-onset group were collected within 12 months of disease onset, when the probability of remission phase is highest. Recently, it was shown that most patients in the age group 12–30 years experience their remission phase within one year after T1D onset and that the HbA1c level after one year was significantly lower in the remitters [37]. Overall, the hypothesis put forward by Wentzell et al. [2] that an early age of T1D onset has a favourable effect on perceived diabetes distress later in life was confirmed.

Because there is some overlap of diabetes distress with depression, we also screened for the presence of depression [5]. The proportion of participants who screened positive for depressive symptoms was in the lower range of the prevalence observed in a recent analysis [38]. We are aware of only one single-centre study from Japan that investigated the prevalence of screening positive for depression (PHQ-9 scores ≥ 10) in adults (mean age 40 years, 69% women) with different ages at T1D onset. The proportions of participants who screened positive for depression were 21% (0–12 years of age at onset), 18% (13–19 years of age at onset) and 13% (20–40 years of age at onset). As in our adjusted analyses, the groups with positive and negative depression screening results did not differ with respect to age at onset [39].

### Possible explanations

From our observations, the question arises as to the possible reasons for the greater diabetes distress in emerging adults with a short diabetes duration. It is reasonable to assume that the disease places a particular burden on this group of patients because the life-changing onset of the disease coincides with other changes during this stage of life. Emerging adulthood is a dynamic and changeable period with unique developmental, social, and emotional challenges [2] that requires specialised diabetes care and education [12]. The complexity of the disease experience is individually different, as are the individual resources and problems related to living with diabetes. Perhaps, young adults with long-term diabetes have been more supported by their parents or significant others than their newly diagnosed peers. Unfortunately, we are not aware of any study results on this. Another aspect is that diabetes can both strengthen and hinder one's own definition of identity [3]. Findings from interviews with female emerging adults indicated that they reframed living with diabetes as an opportunity for empowerment and personal growth, even if they experienced distress [40]. Although the generalisability of this study may be limited due to gender differences in diabetes perceptions [41], letting go of habitual patterns of interpretation regarding the disease can be helpful for all people with diabetes. This process of cognitive reframing often happens naturally and unconsciously, but it can also take place as cognitive restructuring under the guidance of a psychologist [42]. In patients with disease onset in adulthood and, consequently, a short disease duration, this process is unlikely to have progressed to the same extent.

### Implications

Our results imply that the relevant proportion of participants who screened positive for diabetes distress in both the childhood-onset (long-term) and adult-onset (short-term) T1D groups calls for awareness by health care professionals and individual counselling to reduce diabetes distress. Emerging adults with short-term T1D may need more psychosocial and practical support than is usually offered to integrate the disease into their lives. For diabetes researchers, our study may suggest that accounting for age at onset and diabetes duration, respectively, may help explain heterogeneity in the data when psychological factors are examined.

### Strengths and limitations

A strength of the study is that it draws attention to diabetes distress, which is an important outcome from the patient's perspective but has not been adequately studied thus far. Another strength is that the analysed outcomes were based on validated and widely used screening questionnaires. Furthermore, we applied a doubly robust causal inference approach to best match the different samples.

The main limitation is that the GDS and the early-onset cohort had different focuses and were not designed for this research question. Hence, analyses had to be limited to the small number of potential confounders that were matched in both studies. Therefore, we applied a doubly robust causal inference approach that was particularly suitable to account for unrecorded influencing factors and provide unbiased estimates even if one of the models was misspecified (e.g., because not all influencing factors were included). Although inverse probability weighting did not optimally balance the confounders (in particular age) between the two study groups (Additional file 1: Table S3), the additional regression adjustment considered an imbalance of confounders beyond weighting and suggested that the effect estimates were likely to be unbiased. In our analyses, the HbA1c value proved to be a very strong influencing factor. We assumed that the influencing factors associated with the HbA1c value were indirectly considered by the chosen method of analysis. In addition, we cannot rule out the possibility that the comparatively small sample size of one study group had an impact on the results in that the confidence intervals of the estimated differences were enlarged and existing differences may not have been demonstrated to be statistically significant. Another limitation is that both studies were not representative and relied on different methods for participant recruitment: one study was population-based and included persons from all over Germany, while the other study mainly recruited participants from the areas surrounding the seven GDS centres with HbA1c < 9%. The study population of the adult-onset sample from centres (in total n = 36) other than the DDZ (n = 127) was too small to consider cluster effects in the analyses. In addition, it is likely that there was a selection bias in both studies, as people who wanted to address their condition and engage in the advancement of diabetes research were more likely to participate. However, despite the lack of representativeness, an association between age at onset and diabetes distress was evident. Finally, it is not possible to distinguish the influence of the age at T1D onset from the influence of the duration of diabetes from our data.

## Conclusions

Using an innovative analytical approach, our results indicate that emerging adults with recent T1D onset are at higher risk of diabetes distress than those with T1D onset during early childhood. Thus, age at onset and diabetes duration should be reported and considered as possible influencing factors in diabetes-related studies. Emerging adults with short-term T1D are an important group requiring psychosocial support at disease onset, as is usual in childhood onset, and further investigation regarding this topic is needed.

## Supplementary Information

**Additional file1: Figure S1.** Early-onset cohort study. **Figure S2.** Sample selection of the adult-onset study group. **Table S1.** Composition of the adult-onset study group. **Table S2.** PAID-20 items response frequencies. **Table S3.** Standardised difference of confounders between adult-onset and childhood-onset T1D group unweighted and inverse probability weighted

## Data Availability

The datasets generated and/or analysed during the current study are available from the corresponding author upon reasonable request.

## Abbreviations

ACE: Average causal effect
BMI: Body mass index
CI: Confidence interval
DDZ: German Diabetes Center
GDS: German Diabetes Study
HbA1c: Haemoglobin A1c
SD: Standard deviation
T1D: Type 1 diabetes
PHQ-9: 9-item depression module from the full patient health questionnaire
PAID-20: 20-item problem areas in diabetes scale

## References

[1] Arnett JJ (2000). Emerging adulthood. A theory of development from the late teens through the twenties. Am Psychol.

[2] Wentzell K, Vessey JA, Laffel LMB (2020). How do the challenges of emerging adulthood inform our understanding of diabetes distress?. An Integrative Review Curr Diab Rep.

[3] Fioretti C, Mugnaini C (2022). Living with type 1 diabetes mellitus in emerging adulthood: a qualitative study. Br J Health Psychol.

[4] Fisher L, Gonzalez JS, Polonsky WH (2014). The confusing tale of depression and distress in patients with diabetes: a call for greater clarity and precision. Diabet Med.

[5] Kiriella DA, Islam S, Oridota O, Sohler N, Dessenne C, de Beaufort C (2021). Unraveling the concepts of distress, burnout, and depression in type 1 diabetes: a scoping review. EClinicalMedicine.

[6] Snoek FJ, Bremmer MA, Hermanns N (2015). Constructs of depression and distress in diabetes: time for an appraisal. Lancet Diabetes Endocrinol.

[7] Byrne M, O'Connell A, Egan AM, Dinneen SF, Hynes L, O'Hara MC (2017). A core outcomes set for clinical trials of interventions for young adults with type 1 diabetes: an international, multi-perspective Delphi consensus study. Trials.

[8] Stahl-Pehe A, Glaubitz L, Bächle C, Lange K, Castillo K, Tönnies T (2019). Diabetes distress in young adults with early-onset type 1 diabetes and its prospective relationship with HbA1c and health status. Diabet Med.

[9] Joensen LE, Tapager I, Willaing I (2013). Diabetes distress in type 1 diabetes–a new measurement fit for purpose. Diabet Med.

[10] Inverso H, LeStourgeon LM, Parmar A, Bhangui I, Hughes B, Straton E (2022). Demographic and glycemic factors linked with diabetes distress in teens with type 1 diabetes. J Pediatr Psychol.

[11] Déniz-García A, Díaz-Artiles A, Saavedra P, Alvarado-Martel D, Wägner AM, Boronat M (2022). Impact of anxiety, depression and disease-related distress on long-term glycaemic variability among subjects with type 1 diabetes mellitus. BMC Endocr Disord.

[12] Gregory JW, Cameron FJ, Joshi K, Eiswirth M, Garrett C, Garvey K (2022). ISPAD clinical practice consensus guidelines 2022: diabetes in adolescence. Pediatr Diabetes.

[13] Rawshani A, Sattar N, Franzen S, Rawshani A, Hattersley AT, Svensson AM (2018). Excess mortality and cardiovascular disease in young adults with type 1 diabetes in relation to age at onset: a nationwide, register-based cohort study. Lancet.

[14] Baek JH, Lee WJ, Lee BW, Kim SK, Kim G, Jin SM (2021). Age at Diagnosis and the risk of diabetic nephropathy in young patients with type 1 diabetes mellitus. Diabetes Metab J.

[15] Tonoli C, Heyman E, Roelands B, Pattyn N, Buyse L, Piacentini MF (2014). Type 1 diabetes-associated cognitive decline: a meta-analysis and update of the current literature. J Diabetes.

[16] Parviainen A, Härkönen T, Ilonen J, But A, Knip M (2022). Heterogeneity of type 1 diabetes at diagnosis supports existence of age-related endotypes. Diabetes Care.

[17] Lašaitė L, Dobrovolskienė R, Danytė E, Stankutė I, Ražanskaitė-Virbickienė D, Schwitzgebel V (2016). Diabetes distress in males and females with type 1 diabetes in adolescence and emerging adulthood. J Diabetes Complicat.

[18] Lašaitė L, Ostrauskas R, Žalinkevičius R, Jurgevičienė N, Radzevičienė L (2015). Profile of mood states in adult type 1 diabetes mellitus men and women with disease onset in childhood and in adulthood. JPEM.

[19] Kroenke K, Spitzer RL, Williams JBW (2001). The PHQ-9: Validity of a Brief Depression Severity Measure. J Gen Intern Med.

[20] Stahl A, Straßburger K, Lange K, Bächle C, Holl RW, Giani G (2012). Health-related quality of life among German youths with early-onset and long-duration type 1 diabetes. Diabetes Care.

[21] Szendroedi J, Saxena A, Weber KS, Strassburger K, Herder C, Burkart V (2016). Cohort profile: the German diabetes study (GDS). Cardiovasc Diabetol.

[22] Vallis M, Willaing I, Holt RIG (2018). Emerging adulthood and type 1 diabetes: insights from the DAWN2 Study. Diabet Med.

[23] Luyckx K, Rassart J, Aujoulat I, Goubert L, Weets I (2016). Self-esteem and illness self-concept in emerging adults with type 1 diabetes: long-term associations with problem areas in diabetes. J Health Psychol.

[24] Rassart J, Luyckx K, Berg CA, Bijttebier P, Moons P, Weets I (2015). Psychosocial functioning and glycemic control in emerging adults with type 1 diabetes: A 5-year follow-up study. Health Psychol.

[25] Wentzel K, Vessey JA, Laffel L, Ludlow L (2020). Diabetes distress in emerging adults: refining the problem areas in diabetes-emerging adult version using rasch analysis. J Appl Meas.

[26] Polonsky WH, Anderson BJ, Lohrer PA, Welch G, Jacobson AM, Aponte JE (1995). Assessment of diabetes-related distress. Diabetes Care.

[27] Hermanns N, Kulzer B, Krichbaum M, Kubiak T, Haak T (2006). How to screen for depression and emotional problems in patients with diabetes: comparison of screening characteristics of depression questionnaires, measurement of diabetes-specific emotional problems and standard clinical assessment. Diabetologia.

[28] de Joode JW, van Dijk SEM, Walburg FS, Bosmans JE, van Marwijk HWJ, de Boer MR (2019). Diagnostic accuracy of depression questionnaires in adult patients with diabetes: a systematic review and meta-analysis. PLoS ONE.

[29] Levis B, Benedetti A, Thombs BD (2019). Accuracy of Patient Health Questionnaire-9 (PHQ-9) for screening to detect major depression: individual participant data meta-analysis. BMJ.

[30] Kroenke K, Spitzer RL, Williams JB, Lowe B (2010). The patient health questionnaire somatic, anxiety, and depressive symptom scales: a systematic review. Gen Hosp Psychiatry.

[31] Wooldridge JM (2010). Econometric analysis of cross section and panel data.

[32] Bang H, Robins JM (2005). Doubly robust estimation in missing data and causal inference models. Biometrics.

[33] Skinner TC, Joensen L, Parkin T (2020). Twenty-five years of diabetes distress research. Diabet Med.

[34] Hagger V, Hendrieckx C, Cameron F, Pouwer F, Skinner TC, Speight J (2018). Diabetes distress is more strongly associated with HbA1c than depressive symptoms in adolescents with type 1 diabetes: results from diabetes MILES Youth-Australia. Pediatr Diabetes.

[35] Strandberg RB, Graue M, Wentzel-Larsen T, Peyrot M, Rokne B (2014). Relationships of diabetes-specific emotional distress, depression, anxiety, and overall well-being with HbA1c in adult persons with type 1 diabetes. J Psychosom Res.

[36] Van Bastelaar KMP, Pouwer F, Geelhoed-Duijvestijn PHLM, Tack CJ, Bazelmans E, Beekman AT (2010). Diabetes-specific emotional distress mediates the association between depressive symptoms and glycaemic control in type 1 and type 2 diabetes. Diabet Med.

[37] Yazidi M, Mahjoubi S, Oueslati I, Chaker F, Chihaoui M (2022). The remission phase in adolescents and young adults with newly diagnosed type 1 diabetes mellitus: prevalence, predicting factors and glycemic control during follow-up. Arch Endocrinol Metab.

[38] Farooqi A, Gillies C, Sathanapally H, Abner S, Seidu S, Davies MJ (2022). A systematic review and meta-analysis to compare the prevalence of depression between people with and without type 1 and type 2 diabetes. Prim Care Diabetes.

[39] Takaike H, Miura J, Ishizawa K, Babazono T (2022). High prevalence of depressive symptoms among people with pediatric-onset and adolescent-onset type 1 diabetes: a cross-sectional analysis of the diabetes study from the center of Tokyo women’s medical university. J Diabetes Investig.

[40] Kruger S, Deacon E, van Rensburg E, Segal DG (2021). Young adult women’s meaning-making of living with type 1 diabetes: towards growth and optimism. Psychol Health.

[41] Castellano-Guerrero AM, Guerrero R, Ruiz-Aranda D, Perea S, Pumar A, Relimpio F (2020). Gender differences in quality of life in adults with long-standing type 1 diabetes mellitus. Diabetol Metab Syndr.

[42] Robson JP, Troutman-Jordan ML (2014). A concept analysis of cognitive reframing. J Theory Constr Test.

