# Supplementary material for: Comparison of diabetes distress and depression screening results of emerging adults with type 1 diabetes onset at different ages: findings from the German early-onset T1D study and the German Diabetes Study (GDS)
Source: Diabetol Metab Syndr. 2023 Feb 19;15:24. doi: 10.1186/s13098-023-00994-2 (PMC9940340; doi:10.1186/s13098-023-00994-2)
Supplement: Supplementary file 1 — Additional file1: Figure S1. Early-onset cohort study. Figure S2. Sample selection of the adult-onset study group. Table S1. Composition of the adult-onset study group. Table S2. PAID-20 items response frequencies. Table S3. Standardised difference of confounders between adult-onset and childhood-onset T1D group unweighted and inverse probability weighted [file 13098_2023_994_MOESM1_ESM.docx]

**Online-Only Additional file**

**Figure S1** Early-onset cohort study


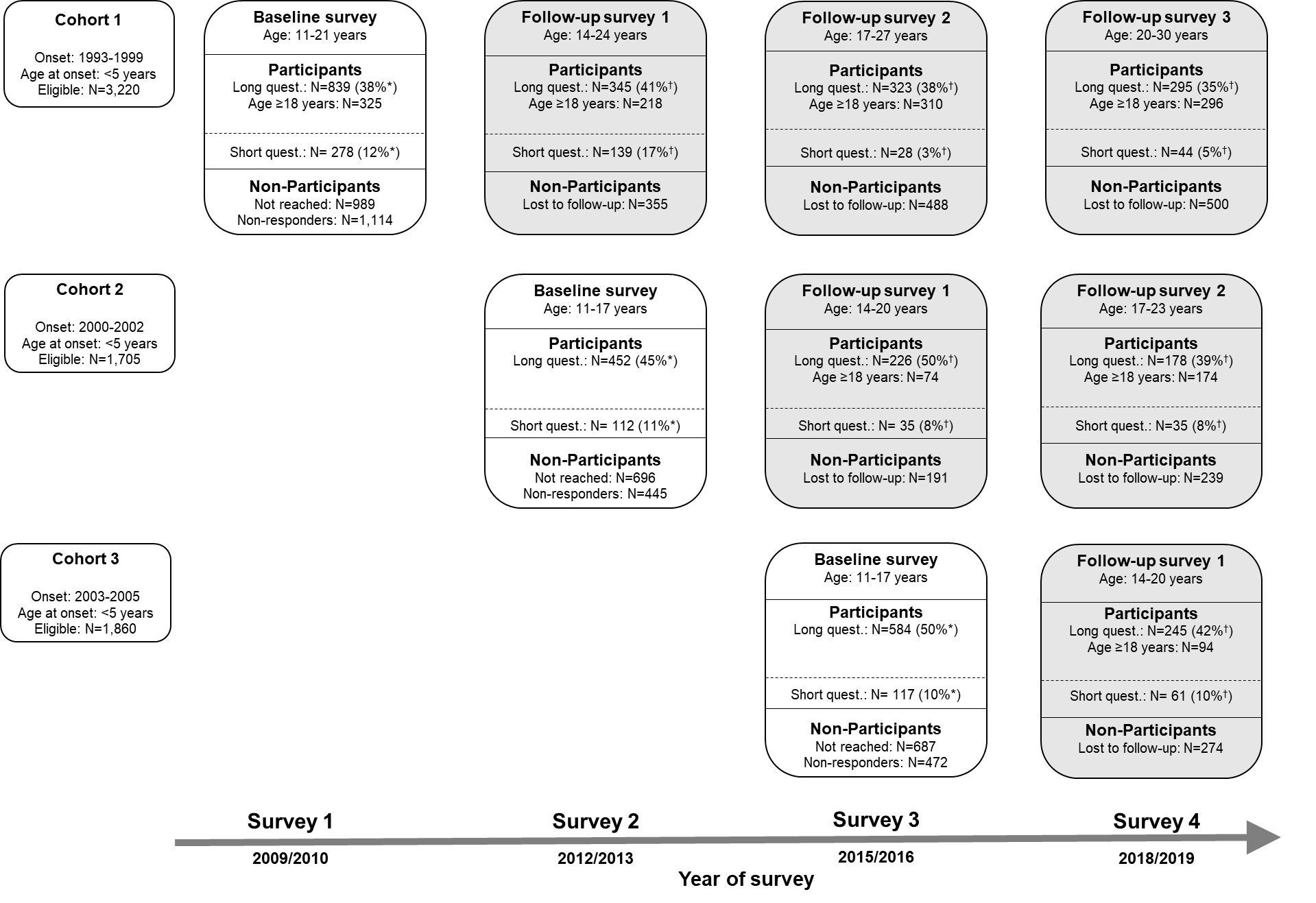


Long quest.: Study participants answered comprehensive questionnaires. Only participants surveyed since the year 2012 aged ≥18 years answered the PAID-20- and PHQ-9-questionnaires. A total of 50 participants from survey 2, 135 participants from survey 3 and 564 participants from survey 4 were included in this investigation.

Short quest.: Some participants answered only key questions of a short questionnaire.

* % of persons invited at baseline (eligible - not reached)

^†^ % of participants at baseline

**Figure S2** Sample selection of the adult-onset study group

**
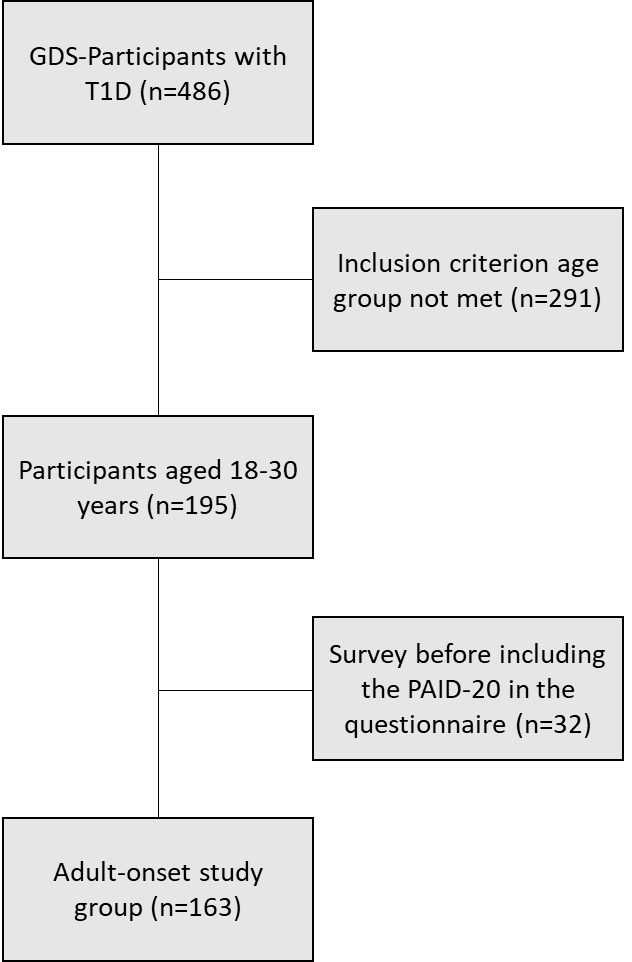
**

**Table S1** Composition of the adult-onset study group

| German Diabetes Study (GDS) participating centers | Number of patients for the analysis | | | |
| --- | --- | --- | --- | --- |
|  | Total | Baseline | 5-year-follow-up | 10-year-follow-up |
| German Diabetes Center, Düsseldorf | 127 | 84 | 32 | 11 |
| German Institute for Human Nutrition / Charité Berlin, Berlin / Potsdam | 4 | 4 | 0 | 0 |
| Tübingen University Hospital, Tübingen | 5 | 5 | 0 | 0 |
| Ludwig-Maximilians-Universität, München | 3 | 3 | 0 | 0 |
| Heidelberg University Hospital, Heidelberg | 12 | 9 | 3 | 0 |
| Dresden University Hospital, Dresden | 0 | 0 | 0 | 0 |
| Leipzig University Hospital, Leipzig | 3 | 3 | 0 | 0 |
| Schleswig-Holstein University Hospital, Lübeck | 9 | 9 | 0 | 0 |

**Table S2** PAID-20 items response frequencies

| **PAID-20 item** | **Study group** | **N** | **Not a problem [%]** | **Minor problem [%]** | **Moderate problem [%]** | **Somewhat serious problem [%]** | **Serious problem [%]** | **P *** |
| --- | --- | --- | --- | --- | --- | --- | --- | --- |
| 1. Not having clear and concrete goals for your diabetes care? | Childhood-onset | 746 | 57 | 29 | 9 | 3 | 2 | 0.585 |
|  | Adult-onset | 151 | 58 | 24 | 11 | 5 | 3 |  |
| 2. Feeling discouraged with your diabetes treatment plan? | Childhood-onset | 747 | 53 | 23 | 12 | 9 | 4 | 0.084 |
|  | Adult-onset | 151 | 50 | 30 | 15 | 5 | 1 |  |
| 3. Feeling scared when you think about living with diabetes? | Childhood-onset | 746 | 50 | 22 | 15 | 9 | 4 | 0.006 |
|  | Adult-onset | 151 | 32 | 33 | 20 | 10 | 5 |  |
| 4. Uncomfortable social situations related to your diabetes care (e.g., people telling you what to eat)? | Childhood-onset | 746 | 49 | 25 | 16 | 6 | 4 | 0.121 |
|  | Adult-onset | 151 | 36 | 32 | 19 | 9 | 4 |  |
| 5. Feelings of deprivation regarding food and meals? | Childhood-onset | 746 | 50 | 31 | 12 | 4 | 3 | <0.001 |
|  | Adult-onset | 151 | 27 | 25 | 30 | 13 | 5 |  |
| 6. Feeling depressed when you think about living with diabetes? | Childhood-onset | 747 | 59 | 21 | 10 | 7 | 4 | 0.084 |
|  | Adult-onset | 151 | 48 | 27 | 16 | 7 | 2 |  |
| 7. Not knowing if your mood or feelings are related to your diabetes? | Childhood-onset | 746 | 41 | 23 | 20 | 11 | 5 | 0.951 |
|  | Adult-onset | 151 | 42 | 25 | 19 | 10 | 4 |  |
| 8. Feeling overwhelmed by your diabetes? | Childhood-onset | 747 | 55 | 26 | 11 | 7 | 2 | 0.585 |
|  | Adult-onset | 151 | 57 | 26 | 13 | 3 | 1 |  |
| 9. Worrying about low blood sugar reactions? | Childhood-onset | 746 | 36 | 35 | 18 | 8 | 4 | 0.464 |
|  | Adult-onset | 151 | 29 | 36 | 19 | 13 | 3 |  |
| 10. Feeling angry when you think about living with diabetes? | Childhood-onset | 747 | 48 | 23 | 13 | 11 | 6 | 0.084 |
|  | Adult-onset | 151 | 40 | 26 | 22 | 8 | 5 |  |
| 11. Feeling constantly concerned about food and eating? | Childhood-onset | 747 | 55 | 25 | 10 | 5 | 4 | <0.001 |
|  | Adult-onset | 151 | 37 | 23 | 23 | 11 | 6 |  |
| 12. Worrying about the future and the possibility of serious complications? | Childhood-onset | 746 | 14 | 25 | 26 | 22 | 14 | 0.951 |
|  | Adult-onset | 151 | 15 | 25 | 25 | 19 | 16 |  |
| 13. Feelings of guilt or anxiety when you get off track with your diabetes management? | Childhood-onset | 745 | 22 | 26 | 28 | 17 | 8 | 0.003 |
|  | Adult-onset | 151 | 33 | 34 | 21 | 7 | 5 |  |
| 14. Not “accepting” your diabetes? | Childhood-onset | 741 | 67 | 15 | 7 | 4 | 7 | 0.084 |
|  | Adult-onset | 150 | 63 | 23 | 8 | 4 | 2 |  |
| 15. Feeling unsatisfied with your diabetes physician? | Childhood-onset | 743 | 76 | 13 | 6 | 2 | 3 | 0.585 |
|  | Adult-onset | 150 | 72 | 16 | 7 | 4 | 1 |  |
| 16. Feeling that diabetes is taking up too much of your mental and physical energy every day? | Childhood-onset | 745 | 46 | 27 | 15 | 8 | 4 | 0.782 |
|  | Adult-onset | 151 | 42 | 26 | 19 | 10 | 3 |  |
| 17. Feeling alone with your diabetes? | Childhood-onset | 745 | 66 | 20 | 9 | 3 | 2 | 0.585 |
|  | Adult-onset | 151 | 65 | 23 | 7 | 2 | 3 |  |
| 18. Feeling that your friends and family are not supportive of your diabetes management efforts? | Childhood-onset | 746 | 81 | 13 | 4 | 1 | 1 | 0.210 |
|  | Adult-onset | 151 | 74 | 19 | 3 | 3 | 1 |  |
| 19. Coping with complications of diabetes? | Childhood-onset | 739 | 69 | 16 | 9 | 4 | 2 | 0.585 |
|  | Adult-onset | 149 | 64 | 20 | 7 | 7 | 2 |  |
| 20. Feeling “burned out” by the constant effort needed to manage diabetes? | Childhood-onset | 744 | 56 | 21 | 13 | 5 | 4 | 0.585 |
|  | Adult-onset | 148 | 54 | 26 | 13 | 6 | 1 |  |

* P value for the comparison of the childhood-onset and adult-onset study group adjusted for multiple-comparison testing per the Benjamini-Hochberg procedure to control for the false discovery rate [Benjamini Y, Hochberg Y. Controlling the False Discovery Rate: A Practical and Powerful Approach to Multiple Testing. Journal of the Royal Statistical Society 1995; 57:289-300.]

**Table S3 Standardized difference of confounders between adult-onset and childhood-onset T1D group unweighted and inverse probability weighted**

| **Outcome** | **Confounder** | **Subgroup** | **Model 1** | | **Model 2** | | **Model 3** | |
| --- | --- | --- | --- | --- | --- | --- | --- | --- |
|  |  |  | StdDiff | Weighted StdDiff | StdDiff | Weighted StdDiff | StdDiff | Weighted StdDiff |
| PAID-20 total score | Sex | Male | -0.278 | -0.005 | -0.274 | -0.062 | 0.274 | 0.062 |
|  | Age | - | 1.278 | -0.159 | 1.248 | -0.180 | 1.248 | -0.181 |
|  | HbA1c | - | - | - | -1.057 | -0.064 | -1.057 | -0.064 |
|  | School-leaving certificate | High school graduation | - | - | - | - | 0.172 | 0.186 |
| Positive screening for diabetes distress | Sex | Male | -0.262 | -0.008 | -0.257 | -0.074 | 0.257 | 0.079 |
|  | Age | - | 1.286 | -0.145 | 1.255 | -0.177 | 1.255 | -0.172 |
|  | HbA1c | - | - | - | -1.059 | -0.066 | -1.059 | -0.064 |
|  | School-leaving certificate | High school graduation | - | - | - | - | 0.199 | 0.168 |
| PHQ-9 total score | Sex | Male | -0.260 | 0.001 | -0.256 | -0.068 | 0.256 | 0.073 |
|  | Age | - | 1.282 | -0.155 | 1.252 | -0.183 | 1.252 | -0.178 |
|  | HbA1c | - | - | - | -1.057 | -0.054 | -1.057 | -0.052 |
|  | School-leaving certificate | High school graduation | - | - | - | - | 0.195 | 0.166 |
| Positive screening for depression | Sex | Male | -0.253 | 0.003 | -0.249 | -0.055 | 0.257 | 0.074 |
|  | Age | - | 1.279 | -0.150 | 1.252 | -0.176 | 1.254 | -0.176 |
|  | HbA1c | - | - | - | -1.049 | -0.049 | -1.059 | -0.053 |
|  | School-leaving certificate | High school graduation | - | - | - | - | 0.194 | 0.165 |

Model 0: not adjusted

Model 1: adjusted for age and sex

Model 2: adjusted for age, sex and HbA1c

Model 3: adjusted for age, sex, HbA1c, school-leaving certificate

StdDiff: standardized difference of confounder between adult-onset and childhood-onset T1D groups

Absolute values of standardized differences less than 0.1 indicate a good balance between groups [Austin PC, Stuart EA. Moving towards best practice when using inverse probability of treatment weighting (IPTW) using the propensity score to estimate causal treatment effects in observational studies. Statistics in medicine 2015; 34:3661-3679.]. Thus, after inverse probability weighting satisfactory balance is indicated for sex, HbA1c and school education, while balance for age is still scarce. In further analysis, however, we additionally applied regression adjustment to consider unbalance of confounders beyond weighting.
